# Increased methane emissions from oil and gas following the Soviet Union’s collapse

**DOI:** 10.1073/pnas.2314600121

**Published:** 2024-03-12

**Authors:** Tai-Long He, Ryan J. Boyd, Daniel J. Varon, Alexander J. Turner

**Affiliations:** ^a^Department of Atmospheric Sciences, University of Washington, Seattle, WA 98195; ^b^John A. Paulson School of Engineering and Applied Sciences, Harvard University, Cambridge, MA 02138

**Keywords:** methane, deep learning, remote sensing, plume detection, fossil fuel

## Abstract

Global atmospheric methane concentrations rose by 10 to 15 ppb/y in the 1980s before abruptly slowing to 2 to 8 ppb/y in the early 1990s. This period in the 1990s is known as the “methane slowdown” and has been attributed in part to the collapse of the former Soviet Union (USSR) in December 1991, which may have decreased the methane emissions from oil and gas operations. Here, we develop a methane plume detection system based on probabilistic deep learning and human-labeled training data. We use this method to detect methane plumes from Landsat 5 satellite observations over Turkmenistan from 1986 to 2011. We focus on Turkmenistan because economic data suggest it could account for half of the decline in oil and gas emissions from the former USSR. We find an increase in both the frequency of methane plume detections and the magnitude of methane emissions following the collapse of the USSR. We estimate a national loss rate from oil and gas infrastructure in Turkmenistan of more than 10% at times, which suggests the socioeconomic turmoil led to a lack of oversight and widespread infrastructure failure in the oil and gas sector. Our finding of increased oil and gas methane emissions from Turkmenistan following the USSR’s collapse casts doubt on the long-standing hypothesis regarding the methane slowdown, begging the question: “what drove the 1992 methane slowdown?”

## Significance

The rate of increase in atmospheric methane concentrations abruptly declined in the early 1990s. This decline in the growth rate is known as the “methane slowdown.” Previous work attributed the methane slowdown to a reduction in oil and gas emissions from the former Soviet Union following its collapse. Our work uses historical satellite imagery and deep learning to quantify the methane emissions in Turkmenistan, a major oil- and gas-producing Soviet republic, from 1986 to 2011. We find widespread methane intensity from oil and gas infrastructure that results in an increase in Turkmen methane emissions following the collapse, casting doubt on the hypothesis that the collapse of the Soviet Union drove the methane slowdown.

Atmospheric methane has exhibited both periods of rapid growth and stabilization since in situ observations began in the early 1980s. There has been much debate about the causes of these variations (1–16). One such variation occurred in the early 1990s when the methane growth rate ($d\left[{\mathrm{CH}}_{4}\right]/dt$) abruptly declined from 10 to 15 ppb/y to 2 to 8 ppb/y in 1992. This change in the methane growth rate is referred to as the “methane slowdown.” Previous work observed a decline in the inter-polar difference (IPD; difference between Arctic and Antarctic methane concentrations) that coincided with the methane slowdown (1, 2, 17). Analysis of stable carbon isotopes of methane ($\delta^{13}$C-CH_4_) suggested a decline in isotopically heavy sources (4) in the early 1990s, such as oil and gas (O&G), or an increase in light sources [e.g., wetlands (18, 19)]. Following this, previous work (1, 2, 4) hypothesized that the collapse of the USSR contributed to the abrupt change in methane growth rate in 1992 due to a decrease in O&G production, resulting in lower methane emissions from a high-latitude source. This hypothesis is compatible with both the constraints from the IPD and $\delta^{13}$C-CH_4_. However, recent work has shown how the IPD is affected by extra-polar emissions and variations in atmospheric transport (20), meaning the IPD may not reflect changes in high-latitude sources as originally hypothesized. Regarding $\delta^{13}$C-CH_4_, there is large overlap in the isotopic source signatures (6, 21) and, as such, they do not unambiguously constrain fossil fuel sources. Uncertainties in historical methane emissions from wetlands and the methane sink further complicate the interpretation (10, 19, 22). Here, we assess the role of the collapse of the USSR on the methane slowdown in 1992 using historical satellite observations.

Analysis of economic data shows a decline in gas production from former USSR republics following the collapse (23). This economic data can be used to construct a “bottom–up” estimate of methane emissions. *SI Appendix*, Fig. S1 shows the O&G production data and a bottom–up estimate of methane emissions for the USSR and Turkmenistan (24). Bottom–up methods predict a decline in methane emissions from USSR O&G of 1.400 Gg/y between 1992 and 1997. Turkmenistan’s O&G emissions are predicted to decline by 700 Gg/y. The severe decline in Turkmen gas production was driven by the decrease and eventual complete cessation of demand from republics in the former USSR, primarily Ukraine, between 1993 and 1998 (25). Bottom–up methods attribute half of the decline in USSR O&G methane emissions to Turkmenistan, suggesting that it was a particularly important contributor to the methane slowdown in 1992. As such, quantifying historical changes in O&G methane emissions in Turkmenistan is crucial for understanding the drivers of the methane slowdown.

Recent work from Varon et al. (26) demonstrated how land surface imaging satellites can be used to detect and quantify methane emissions from large point sources. Briefly, these satellites have bands in the shortwave infrared (SWIR) that cover methane absorption features near 1.6 and 2.2 μm. The high spatial resolution of these land surface imaging satellites (20–30 m) results in a high signal-to-noise ratio in the vicinity of large methane point sources. This has been used in a number of recent studies (26–28) to quantify methane emissions from O&G operations over the past few years using Landsat 8–9 and Sentinel-2A/B. Landsat 4–5 were the first in the Landsat series to include SWIR bands, potentially allowing the quantification of historical methane plumes. Landsat 5 launched in March 1, 1984, and operated until June 5, 2013. The historical records from Landsat 4–5 may provide new insights into the drivers of variations in atmospheric composition over the past half century.

Here, we develop a methane plume detection system based on an ensemble of deep learning models and trained using human-labeled methane plume masks. This plume detection system is then applied to the 26-y record from Landsat 5 over Turkmenistan. We quantify the point source methane emissions from O&G operations in Turkmenistan before and after the collapse of the USSR. Through comparison with economic data, we estimate a national loss rate from O&G operations in Turkmenistan.

## Detection of Methane Sources in Turkmenistan

**Fig. 1.**
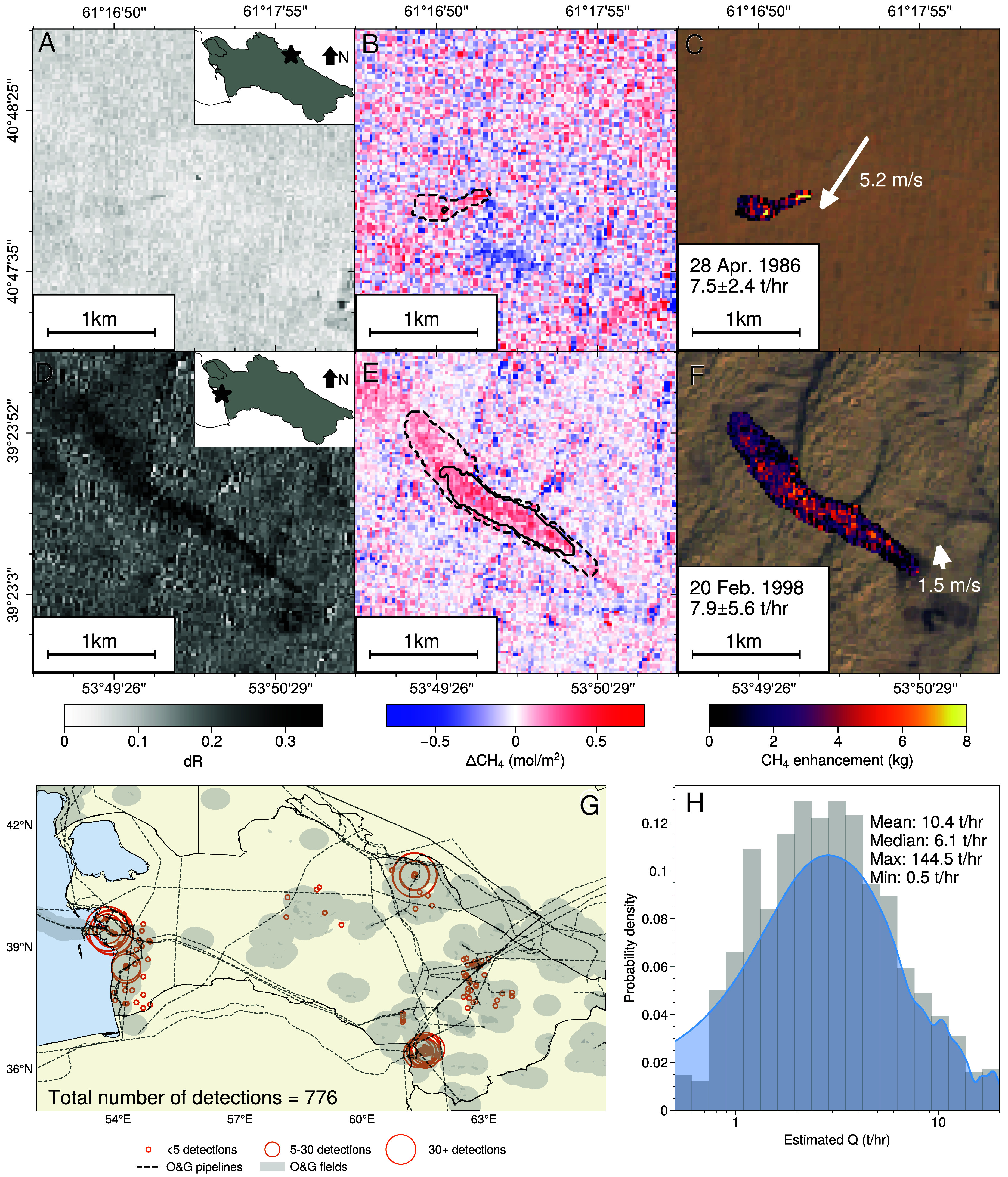
**Detection of methane plumes in Turkmenistan from 1986 to 2011.** (*A*–*C*) Fractional differences in SWIR top-of-atmosphere reflectances (dR), retrieved methane column anomalies, and estimated methane enhancements, respectively, for one of the oldest methane plumes detected in Turkmenistan from Landsat 5. (*D*–*F*) Same as panels (*A*–*C*), but for another methane plume. Dashed plume contours are with low confidence levels and solid contours are for the high confidence regions. (*G*) Location of detected methane plumes. (*H*) Histogram of the methane emissions for the detected plumes.

The 1986 to 2011 Landsat 5 operational period provides data both before and after the collapse of the USSR. Methane plumes were detected over Turkmenistan using the ensemble deep-learning model (*Materials and Methods*), and emissions (*Q*) were quantified using the integrated methane enhancement (IME) method (26, 29, 30). Fig. 1 shows two examples of methane plumes detected in Turkmenistan. Plume detections are based, in part, on the normalized difference in top-of-atmosphere reflectance in the two SWIR bands (dR), similar to other normalized difference indices used in land surface imaging work. We then use a radiative transfer model (26, 30) to determine the methane column anomalies needed to reproduce the observed dR. Fig. 1 *A* and *D* show the dR; Fig. 1 *C* and *F* show theassociated methane column anomalies. The ensemble deep-learning method allows us to calculate regions of high and low confidence in the detected plumes, indicated by the contours in Fig. 1 *B* and *E*. We define our high (low) confidence region as pixels that are classified as a methane plume by more than 75% (10%) of the deep learning ensemble models. Methane emissions for the plumes are then computed using the IME method with the methane anomalies from Landsat 5, plume masks from the plume detection method, and reanalysis windspeed data from the ECMWF Reanalysis v5 (31). Application of this method to automatically detect plumes and quantify emissions with noisy data from the older series of Landsat instruments (4–5) required a number of developments (*Materials and Methods*). To our knowledge, the methane plume shown in the top row of Fig. 1, from 1986, is the oldest methane plume ever observed from space.

Fig. 1*G* shows the location of all detected plumes from Landsat 5. In total, we detected 776 plumes between 1986 and 2011. Each plume was manually examined after detection to evaluate the robustness of the methodology and minimize false detections. Three prominent clusters of plumes can be seen in the southeast, northeast, and in the west along the Caspian Sea. These regions all have extensive O&G operations. Many of these regions have been noted by previous work using instruments on modern satellites: Sentinel-5P (32), Sentinel-2A/B (28, 33), and Landsat 8 (33). We observe intermittent plumes along pipelines in the central and eastern O&G fields in Turkmenistan. To our knowledge, these are some of the first methane plume detections in these regions. Fig. 1*H* shows the statistics of all the detected plumes. The distribution of methane emissions is lognormally distributed with a mean (median) emission rate of 10.4 t/h (6.1 t/h). A lognormal distribution of methane emissions is consistent with previous work characterizing the distribution of methane emissions from O&G operations (29, 32, 34) due to the importance of super-emitters in the methane budget (35). The largest source observed was 145 ± 36 t/h and the smallest source was 0.6 ± 0.2 t/h, representing our best estimate of a detection limit.

## Persistent Methane Emissions from a Single Gas Field

**Fig. 2.**
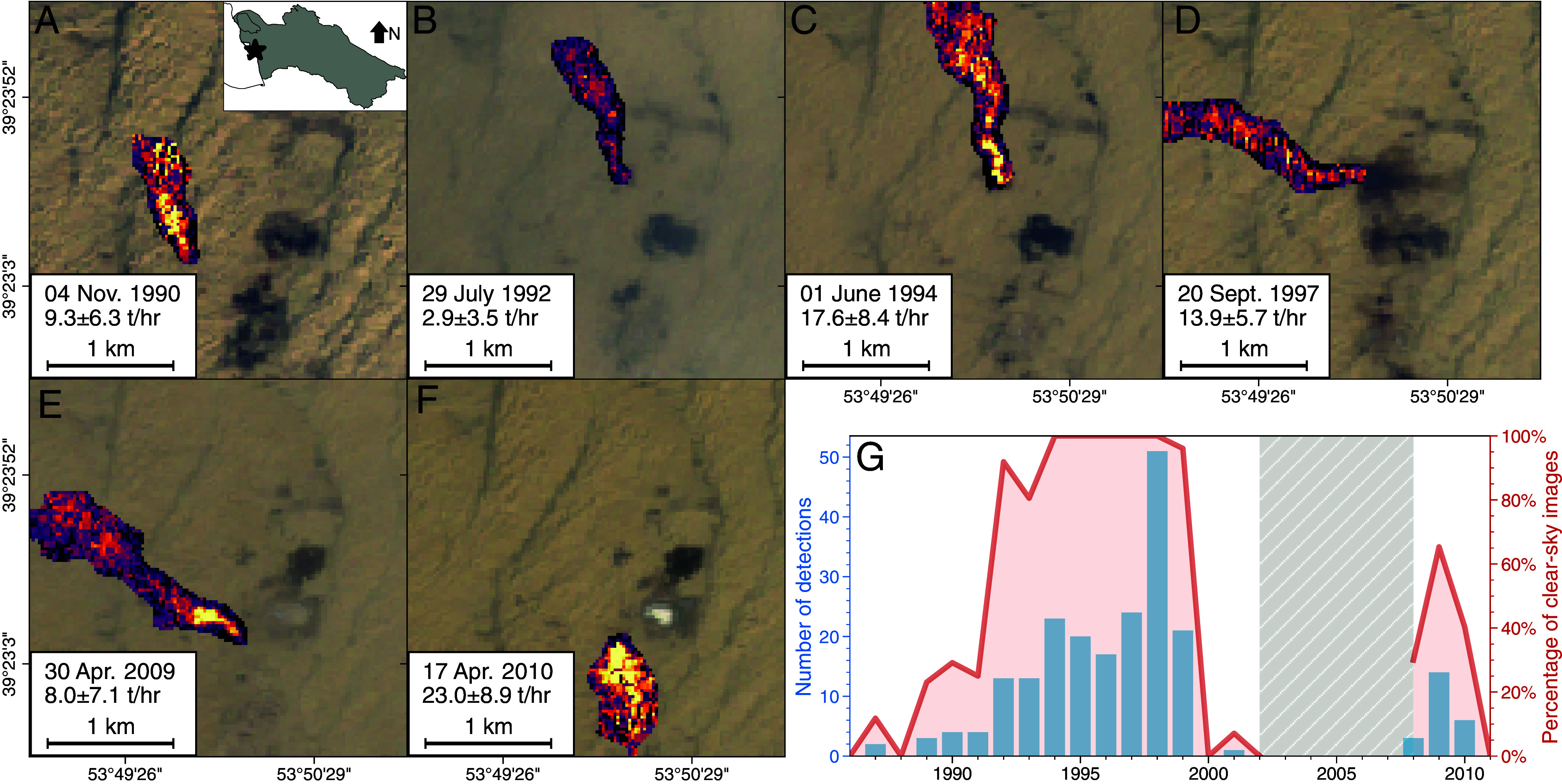
Persistent regional methane emissions from the Barsagelmez Oil Field. (*A*–*F*) Example methane plumes from the Barsagelmez Oil Field (39.391^°^N, 53.833^°^E) from 1986 to 2011. (*G*) Number of detections (light blue) and percent of clear-sky scenes with detections (orange). The gray-shaded area indicates years with no Landsat 5 images available on Google Earth Engine due to the decentralized handling and distribution of Landsat 5 datasets.

Examination of the detected methane plumes shows persistent methane emissions. Fig. 2 shows methane plumes detected in a subregion within the Barsagelmez Oil Field (39.391^°^N, 53.833^°^E) near the Caspian Sea. We first observe methane plumes in 1987. With the exception of 1986, 1988, 2000, and 2002, we observe large methane plumes in this subregion nearly every year data are available. Specifically, we observe methane plumes emanating from three distinct locations within this subregion.

Fig. 2*G* shows the percentage of clear-sky scenes over this subregion that include a methane plume. Prior to the collapse of the USSR in 1991, we observe methane plumes in 0 to 20% of the clear sky scenes between 1986 and 1991. After the collapse, we observe methane plumes in 80 to 100% of the clear sky scenes between 1992 and 1999. This sharp increase in the frequency of plume detections coincides with the decline in Turkmenistan gas production starting in 1992 (*SI Appendix*, Fig. S1). From 1994 to 1999, we observe a methane plume in more than 95% of the clear sky scenes. In other words, we observe 6 y of nearly continuous methane emissions from a single source. The start of these continuous methane emissions follows Russia’s refusal to allow Turkmenistan to pass gas through Russian pipelines to Europe in 1994 (36, 37). The situation was observed to improve in 2000 with only a single plume detected between 2000 and 2002. The frequency of plume detections increased again from 30% to 66% from 2008 to 2009 before being mitigated in 2011. Turkmen gas production declined in 2009 and 2010 due to the global financial crisis.

We calculated cumulative methane emissions from this subregion within the Barsagelmez Oil Field (*SI Appendix*, Fig. S7*G*). From 1986 to 1992, the cumulative emissions increased at an average rate of 13.4 Gg per year. Beginning in 1992, when the persistent source was detected, the cumulative emissions increased by 80.1 Gg per year through 1999. Ultimately, we observe 0.73 ± 0.13 Tg of methane released from this subregion between 1986 and 2000. There is a data gap from 2002 to 2008 with no Landsat 5 images available due to the decentralized handling and distribution of historical Landsat datasets (38, 39). The point sources detected from 2008 to 2011 add an additional 0.09 Tg, resulting in a lower bound on cumulative emissions of 0.82 ± 0.16 Tg for this subregion from 1986 to 2011 (with missing data from 2002 to 2007). The total amount of methane released from the subregion is equivalent to a 0.30-ppb increase in the steady state atmospheric methane mixing ratio if it were instantaneously released, using a conversion factor (40) of 2.75 Tg CH_4_ ppb^−1^. The contribution to global mean methane concentrations is disproportionately large for just one subregion, indicating an important role of persistent point sources in the methane budget.

## National Emission Estimates from Turkmenistan

**Fig. 3.**
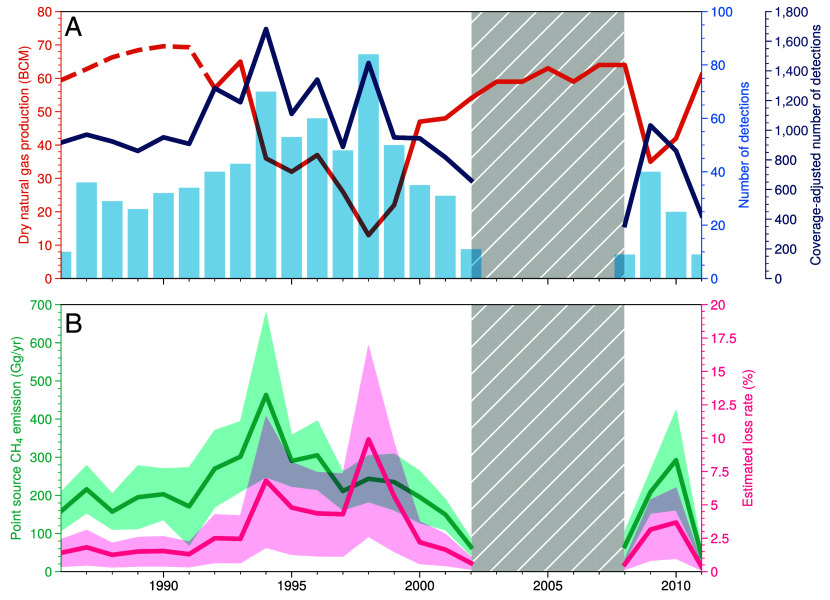
Time series analysis of methane point sources detected in Turkmenistan. (*A*) Number of detected methane plumes per year (light blue), the EIA dry natural gas production (23) (orange), and coverage-adjusted number of detections (dark blue). The dashed orange line between 1986 and 1991 indicates dry natural gas production estimated based on scaling using EDGAR O&G emissions. (*B*) Estimated annual methane emissions from the point sources (teal) and estimated national O&G loss rate in Turkmenistan (pink). The gray-shaded area indicates years with no Landsat 5 images available on Google Earth Engine due to the decentralized handling and distribution of Landsat 5 datasets.

Fig. 3*A* shows the number of methane plume detections over Turkmenistan during the Landsat 5 observational period from 1986 to 2011. To account for the intermittent sampling and variations in cloud cover, we define the expected number of plume detections given perfect sampling as the coverage-adjusted detections: $p_{C}\equiv p_{L}\times n_{I}/n_{L}$, where $p_{L}$ is the number of plumes detected annually, $n_{L}$ is the number of clear-sky scenes in a year, and $n_{I}$ is the number of possible Landsat scenes over Turkmenistan in a year. Prior to the collapse of the USSR, we find 800 to 1,000 coverage-adjusted plumes per year (∼2.5 plumes/d). Both the number of detections and the coverage-adjusted detections increase in 1992 following the collapse of the USSR with the coverage-adjusted plumes increasing by 29% to an average of 1,230 plumes per year (3.4 plumes/d) between 1992 and 1999 with a maximum of 1,600 plumes in 1994 (4.4 plumes/d).

Both the number of detected plumes and the coverage-adjusted plume detections are anti-correlated with the Turkmen natural gas production. After the USSR collapse, the dry natural gas production in Turkmenistan declined 77% from 57 billion cubic meters (BCM) in 1992 to the minimum of 13 BCM in 1998. We detected 84 methane plumes in Turkmenistan in 1998, the most of any year in the Landsat 5 record, when the Turkmenistan dry gas production was at a minimum. 1994 marked the maximum in the coverage-adjusted plume detections and, as mentioned above, Russia began refusing to allow Turkmenistan to pass gas through Russian pipelines to other markets in 1994 (25). We also observe an increase in plume detections in 2009 to 2010. This increase is coincident with a decline in Turkmen dry gas production following the global financial crisis in 2008.

One hypothesis for the increase in methane plume detections in the 1990s is that the socioeconomic decline following the USSR collapse reduced the frequency of maintenance and oversight, increasing the methane loss from O&G operations. To assess this, we calculated methane emissions from each detected plume and estimated O&G loss rates (methane emitted per dry gas production) from 1986 to 2011. Extending the analysis from detected plumes to a national O&G emission estimate requires three assumptions: i) The statistics of the detected plumes are consistent with the true plume frequency, ii) the percent of O&G emissions coming from point sources is invariant, and iii) the point source emissions covary with national O&G emissions in Turkmenistan. The first assumption is necessitated by the low revisit frequency of Landsat 5 (∼3 times per month), meaning that we do not detect all methane plumes. The latter assumption is because the detection limit of Landsat 5 precludes observing methane plumes smaller than 0.5 t/h, meaning there are many O&G sources we do not detect. Following this, we compute the coverage-adjusted point source emissions by scaling the annual methane emissions from detected plumes by the ratio of the maximum possible Landsat scenes in a year to the number of clear-sky scenes. This yields an annual estimate for the point source emissions from Turkmenistan. To account for the sources below our detection limit, we compare our point source emissions to a bottom–up inventory prior to the USSR collapse. This allows us to determine the percent of O&G emissions our method can detect (*SI Appendix*). The average emissions from point sources prior to the collapse was 183.4±22.6 Gg/y, which is ∼18% of the bottom–up O&G emissions for Turkmenistan (24). Our point source emissions are scaled based on the average ratio between the bottom–up O&G emissions and the point source emissions between 1986 and 2000. We compute a lower bound assuming no scaling (i.e., the observed point source emissions represent all the O&G emissions) and the upper bound uses the largest ratio between 1986 and 2000. Finally, we assume the observed point source emissions covary with the national O&G emissions in Turkmenistan.

Fig. 3*B* shows the point source emissions and national gas loss rate in Turkmenistan over the Landsat 5 observational period. Point source emissions from O&G in Turkmenistan were ∼180 Gg/y from 1986 to 1991. The emissions nearly triple to 463.2 ± 215.7 Gg/y in 1994 and remain elevated through 1998 before declining to an average of 136.6 ± 43.4 Gg/y from 2000 to 2002, similar to the pre-collapse level. The national loss rate in Turkmenistan was stable from 1986 to 1991 at 1 to 2%. This loss rate is consistent with previous estimates of methane loss rates based on measurements along pipelines in Russia (41, 42) and is comparable to many O&G basins in the United States (43, 44); other studies suggest even higher loss rates for the USSR than observed here (45). The loss rate exhibits a near-step change increase beginning in 1994 with a maximum of 10% in 1998. Upper bounds on the loss rate in 1994 and 1998 were 12% and 17%, respectively. The average loss rate from 1994 to 1998 was 6%, 4 times larger than the average pre-collapse loss rate. As with the detections, the loss rate is anti-correlated with the dry gas production throughout the record. We also observe an increase in the emissions and loss rate following the financial crisis in 2008.

## Implications for the Methane Budget

Our work finds an anti-correlation between the dry gas production and methane emissions from O&G operations in Turkmenistan from 1986 to 2011. While the focus of our analysis was on Turkmenistan, the work likely has implications for the broader USSR as bottom–up inventories attribute half of the change in USSR emissions to Turkmenistan. We observe an increase in methane plume detections, O&G emissions, and the loss rate from Turkmenistan O&G in 1992 after the collapse of the USSR. The two maximum loss rates occur in 1994 and 1998. These maxima coincide with geopolitical and economic events during this period of turmoil: Russia began refusing to transmit Turkmen gas to other markets in 1994 and Turkmenistan’s dry gas production was at a minimum in 1998. Our results suggest that the socioeconomic turmoil following the USSR collapse resulted in widespread infrastructure failure, large methane loss from O&G operations, and an increase in methane emissions in the 1990s. Given our findings and the outsized role of Turkmenistan in the former USSR O&G emissions budget, we question the previous hypothesis that decreased O&G emissions from the former USSR contributed to the methane slowdown in 1992. Our results beg the question *“what drove the methane slowdown in the 1990s?”*

## Materials and Methods

In this study, we trained an ensemble of deep learning models to detect methane plumes and predict plume masks from images sampled by Sentinel-2 and Landsat satellites. All the models are trained using human-annotated plume masks labeled following the literature. The ensemble is used to search for historical methane plumes in Landsat 5 datasets over Turkmenistan. The plume masks predicted by the ensemble are used to quantify methane emission rates using the IME method. Uncertainties on the estimated flux rates are calculated and provided.

### Deep Learning Model.

The deep learning model we use is adapted from the U-net model, which was originally proposed for biomedical segmentation problems (46). The U-net model has been recently widely applied in the field of earth science (47–50). The schematic diagram of the model architecture is shown in *SI Appendix*, Fig. S2. The U-net model is an encoder–decoder and is constructed based on the convolutional neural networks (CNN) (51). The first half of the model is an encoder, in which the vectors of input information are filtered by two-dimensional kernels in each convolutional layer and the dimensions of the intermediate outputs (also called latent vectors) are reduced by max-pooling layers. The second half of the model is a decoder that up-samples the compressed latent vectors to the model output layer. The up-sampling process is done via convolutional layers and transposed convolutional layers. During the training process, the model predictions are compared against the ground truth, and the differences between the truth and model predictions are used to calculate partial gradients to optimize the convolutional kernels in the model. Compared to the classic U-net model, we replaced the encoder half with the ResNeXt-50 model, which is a more efficient model to extract patterns from images (52). We applied transfer learning by using the pre-trained ResNeXt-50 model weights before each training process to boost the convergence of training and improve final model performance.

### Input and Output Variables.

Methane absorbs strongly around the 1.6 and 2.2 μm bands in the SWIR, which is measured by Landsat 4–9 and Sentinel-2A/B satellites. We hereafter denote the measured reflectance in the 1.6 and 2.2μm bands as $R_{11}$ and $R_{12}$, respectively, following the Sentinel-2 convention. Following the Multi-Band-Single-Pass method (26), we define the following quantity, dR, to capture methane enhancements:

$$\begin{array}{c} dR=\frac{cR_{12}-R_{11}}{R_{11}}, \end{array}$$

where *c* denotes the scaling factor to account for the overall brightness difference between the two bands. The dR quantity could be used for the retrieval of methane concentrations by fitting a radiative transfer model (26, 53).

The input variables for the deep learning model include dR, an estimate of the background dR, the gray-scale RGB image, the normalized difference vegetation index (NDVI), and two ΔdR fields representing differences between dR and the background dR. NDVI is a classic remote sensing index capturing vegetation on land surface, which is defined as follows:

$$\begin{array}{c} \mathrm{NDVI}=\frac{R_{\mathrm{NIR}}-R_{\mathrm{red}}}{R_{\mathrm{NIR}}+R_{\mathrm{red}}}. \end{array}$$

Here, $R_{\mathrm{NIR}}$ and $R_{\mathrm{red}}$ denote the measured reflectance in the near-infrared (NIR) and red bands. The background dR is estimated by averaging dR with structural similarity indices (SSIM) higher than 0.5 within ±180 d from the target scene. SSIM is a metric used frequently in computer vision to measure the similarity between two images, which accounts for image texture and is indicative of the perceived similarity. SSIM is defined by the following equation:

$$\begin{array}{c} SSIM=\frac{\left(2\mu_{x}\mu_{y}+{\left(k_{1}L\right)}^{2}\right)\left(2\sigma_{\mathrm{xy}}+{\left(k_{2}L\right)}^{2}\right)}{\left(\mu_{x}^{2}+\mu_{y}^{2}+{\left(k_{1}L\right)}^{2}\right)\left(\sigma_{x}^{2}+\sigma_{y}^{2}+{\left(k_{2}L\right)}^{2}\right)}, \end{array}$$

where $\mu_{i}$ and $\sigma_{i}$ stand for the mean and SD of the pixels of the corresponding images, respectively. $\sigma_{\mathrm{xy}}$ is the covariance between the two images. $k_{1}=0.01$, $k_{2}=0.03$, and $L=2^{bits\;px^{-1}}-1$ are variables for the stabilization of the index. The first ΔdR field, $\Delta dR_{1}$, is defined by the following equation:

$$\begin{array}{c} \Delta dR_{1}=Z\left(dR-c^{\prime }dR_{\mathrm{bg}}\right), \end{array}$$

where $c^{\prime }$ adjusts the brightness difference between the target scene and the background scene, and *Z* stands for the standard score calculation. The second ΔdR field, $\Delta dR_{2}$, is the Z-score of the difference between dR of the target scene and the raw background dR.

The output of the deep learning model is binary masks of methane plumes. We use human-annotated plume masks, following the literature, using a customized graphical user interface (GUI). *SI Appendix*, Fig. S3 shows the panel of the GUI. Each methane plume was annotated by more than one person, which is helpful for preventing overfitting data from a single labeler. The data labeling was done following the literature about reported recent detected methane plumes. Overall, we labeled 663 methane plumes as the positive dataset, and we labeled 969 satellite scenes without any plumes as the negative dataset. These numbers are low for data-driven methods, so we applied augmentation steps to increase the volume of training dataset. As shown in *SI Appendix*, Fig. S4, the augmentation steps include 90^°^ rotation, horizontal and vertical flip, and addition of 10% Gaussian noise. These augmentation steps are randomly applied for each augmented data sample. We add 10% Gaussian noise to improve the robustness of deep learning models against the noise in Landsat 5 datasets. As shown in *SI Appendix*, Fig. S6, the final training set contains 3,313 positive samples and 4,831 negative samples after the augmentation process.

### Training Details and Construction of the Ensemble.

The loss function we use during the training process is a multi-term loss, which is defined as follows:

$$\begin{array}{c} L=-\sum_{i}\left(y_{i}\ln{\hat{y}}_{i}+\left(1-y_{i}\right)\ln\left(1-{\hat{y}}_{i}\right)\right))+\left(1-\frac{2|Y\cap \hat{Y}|}{\left|Y|+\right|\hat{Y}|}\right). \end{array}$$

Here, $y_{i}$ and ${\hat{y}}_{i}$ represent true labels and predicted labels for each pixel, respectively. *Y* and $\hat{Y}$ stand for the whole set of true labels and predicted labels, respectively. The first term is the binary cross-entropy (BCE) loss, which is popularly used in binary classification problems and is derived by maximizing the likelihood of correctly predicting the binary labels. The second term is the loss from the Dice score. The Dice score calculates the fraction of the overlap between the two sets of true labels and predicted labels over both sets, which measures the overall correctness of mask segmentation. We use the Adam optimization algorithm for the convergence of the training. Training of deep learning model is conducted using NVIDIA A2 Tensor Core GPUs.

Instead of one model, we trained 20 realizations of the deep learning model. The application of ensemble of deep learning models is useful for the quantification of uncertainties in the predicted methane plume masks (54). As shown in the schematic diagram in *SI Appendix*, Fig. S5, each deep learning model is trained using a subset of the training dataset. During the training of each ensemble member, the hyper-parameters associated with training are randomly perturbed. Details about the hyper-parameters are shown in *SI Appendix*, Table S1.

### Quantification of Methane Emission Rates and Uncertainty.

We use the IME method to quantify emission rates of the detected methane point sources (30) and the uncertainties. The flux rate of a point source could be estimated using the following equation:

$$\begin{array}{c} Q=\frac{U_{\mathrm{eff}}}{L}\sum_{j=1}^{N}\Delta \Omega_{j}A_{j}, \end{array}$$

where $U_{\mathrm{eff}}$ is the effective wind speed, *L* is the plume size, $\Delta \Omega_{j}$ represents the methane enhancement in each pixel, and $A_{j}$ stands for the area of pixels. We estimate *L* to be square root of the area of the plume mask. The effective wind speed is calculated using the empirical relationship between $U_{\mathrm{eff}}$ and 10-m wind speed (26):

$$\begin{array}{c} U_{\mathrm{eff}}=\alpha U_{10m}+\beta , \end{array}$$

where α=0.33 and β=0.45ms^−1^.

The uncertainty on *Q* is estimated using the following equation:

$$\begin{array}{c} \delta Q≃\sqrt{{\left(\frac{Q\delta U_{\mathrm{eff}}}{U_{\mathrm{eff}}}\right)}^{2}+{\left(\frac{Q\delta L}{L}\right)}^{2}+{\left(\frac{U_{\mathrm{eff}}\delta \Delta \Omega_{j}}{L}\sum_{j=1}^{N}A_{j}\right)}^{2}}. \end{array}$$

To propagate the uncertainty on *Q*, we use an absolute error of 2 m/s for $U_{10m}$ and a 20% uncertainty on the plume size $(\sum_{j}A_{j})$. For the uncertainty on pixel-wise methane enhancement, we estimate $\delta \Delta \Omega_{j}$ to be the SE calculated using all pixels outside the plume mask.

We acknowledge that there could exist additional uncertainties in Landsat 5 reflectance measurements. For example, land surface changes could lead to changes in surface albedo and roughness. Higher reflectances could lead to larger dR fields, causing positive biases in the estimated methane emissions. However, the biases have minor magnitudes as compared to methane enhancements caused by plumes from point sources. The impact of land surface-caused biases is also mitigated by our method of estimating background methane fields by averaging satellite scenes with a ±180 d time window.

## Supplementary Material

Appendix 01 (PDF)

## Data Availability

The deep learning model was implemented using PyTorch (https://pytorch.org/). The code to reproduce results in this paper is available at https://doi.org/10.5281/zenodo.10491722. The list of detected plumes, the human-labeled plume masks, the optimized model weights for the ensemble system, methane retrievals, and plume masks have been deposited in the Dryad Data Repository (https://doi.org/10.5061/dryad.4mw6m90hp) (55). The methane plume annotation tool is deposited and available at https://doi.org/10.5281/zenodo.10064968. The annotation of methane plumes involves multispectral top-of-atmosphere (TOA) reflectance measurements from Landsat 8 and Sentinel-2, which are available publicly from Google Earth Engine (GEE, https://developers.google.com/earth-engine/datasets). Landsat 5 TOA reflectance measurements are also from Google Earth Engine, which can be accessed using the GEE Python API (https://developers.google.com/earth-engine/tutorials/community/intro-to-python-api). Dry natural gas production data could be accessed from the U.S. Energy Information Administration (EIA) website (https://www.eia.gov/international/data/country/TKM/natural-gas/dry-natural-gas-production). The Emissions Database for Global Atmospheric Research (EDGAR) version 7.0 GHG emission inventory could be accessed from: https://edgar.jrc.ec.europa.eu/dataset_ghg70.
