# Supplementary material for: Increased methane emissions from oil and gas following the Soviet Union’s collapse
Source: Proc Natl Acad Sci U S A. 2024 Mar 12;121(12):e2314600121. doi: 10.1073/pnas.2314600121 (PMC10963001; doi:10.1073/pnas.2314600121)
Supplement: Supplementary file 1 — Appendix 01 (PDF) [file pnas.2314600121.sapp.pdf]

Supporting Information for

Increased methane emissions from oil and gas following the Soviet Union's collapse

Tai-Long He, Ryan J. Boyd, Daniel J. Varon, and Alexander J. Turner

Tai-Long He and Alexander J. Turner.
E-mail: taihe@uw.edu, turneraj@uw.edu

This PDF file includes:

Supporting text
Figs. S1 to S8
Table S1
SI References

Supporting Information Text

This supplementary information includes 7 additional figures and a table discussed in the main text. Fig. S1 shows EDGAR methane emissions associated with O&G productions, compared between the USSR and Turkmenistan. Fig. S2 shows the schematic diagram of the U-net model to predict methane plume mask using Landsat 5 multi-spectral images. Fig. S3 shows the panel of the graphical user interface (GUI) used for labelling methane plumes from the literature. Fig. S4 illustrates the augmentation steps applied to enrich the training data set. Fig. S5 shows the construction and the application of the ensemble of U-net models. Table 1 shows the hyperparameters associated with the training of each ensemble member. Fig. S6 shows the statistics about the original human-labelled training data set and the augmented data set. Fig. S7 shows the same figure as Fig. 2 in the main text with an additional line for the cumulative emissions. Fig. S8 shows the comparison between reported flux rates from (1–3) and the corresponding flux rates estimated by our system.

Limitations of the work. As a result of the limited number of annotated plumes available in the literature, we sometimes have false positive plumes caused by surface features and currently the workflow still involves some human labor. We manually check each detected methane plume to ensure the validity of the detections reported in this study, using the graphical user interface (GUI) tool shown in Fig. S3. Specifically, we use the GUI to visualize and compare the satellite scenes with detections to other satellite overpasses in the months before and after the detection dates. We check if the detected methane plumes are consistent surface features or changing along with the direction of the ERA5 winds (indicated by the arrow on top-right of the panel in Fig. S3).

In the future, to reduce the amount of work from human expertise in removing false detections, additional work could be done to further improve the deep learning model's robustness against noise. Possible solutions include using simulated methane plumes using an idealized model overlaying background surface features from real satellites. The performance of the deep learning model could also benefit from repeated training with additional data sets after more methane plumes are annotated in the future.

Assumptions made for the calculation of loss rates. Several assumptions are made in the calculation of loss rates. We convert the number of point source detection to coverage-adjusted numbers for each location, by multiplying the fraction of detection days (defined as number of overpasses with detections divided by the total number of clear-sky overpasses) with the number of days per year. We assume that cloud coverage does not introduce bias to the estimated likelihood of detection. We estimate annual mean methane emissions from O&G point sources by multiplying the mean estimated flux rates with the coverage-adjusted number of detections, assuming a statistically invariant percent of O&G emissions coming from point sources. We calculate loss rates as the ratio of the point source emissions divided by the gas production data, assuming the validity of the reported gas production data.

We acknowledge that large uncertainties are associated with the estimated loss rates. Historical bottom-up emissions are not as well constrained as modern periods and are often underestimated. We use the multiyear average ratio between point source emissions and bottom-up emissions to mitigate the potentially inconsistent trends in the bottom-up inventory.

The loss rates are estimated using dry natural gas production data, which would be higher than the estimates calculated using gross natural gas production data. There could be missed detections of methane plumes below the detection limit and due to the low sampling frequency of Landsat 5. As a result, the actual loss rates could be different from our estimates. However, this study is based on the best knowledge about methane emissions and natural gas production in Turkmenistan in the 1990s. The number of detected methane point sources is observed to increase in 1991 before and after cloud coverage is accounted for, which may reflect the socioeconomic turmoil in Turkmenistan caused by the USSR's collapse.

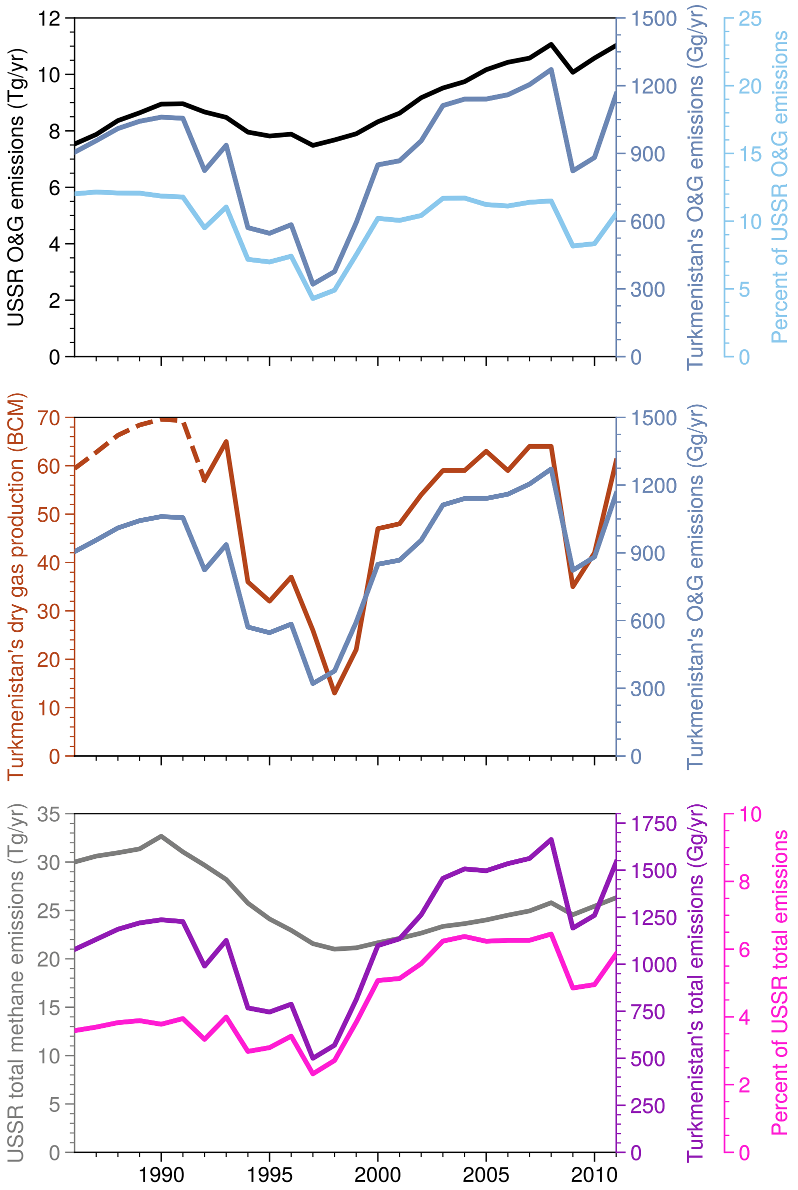

Fig. S1. Variations in EDGAR methane emissions and Turkmenistan dry natural gas production. (Top) EDGAR methane emissions from O&G production for the former USSR (black) and Turkmenistan (dark blue). Turkmenistan's percent of the USSR methane emissions from O&G production is shown in light blue. (Middle) Dry gas production in Turkmenistan (red) and the EDGAR O&G methane emissions for Turkmenistan (dark blue). The dashed line between 1986 and 1991 indicates dry natural gas production estimated based on scaling using EDGAR O&G emissions. (Bottom) EDGAR total methane emissions for the former USSR (gray) and Turkmenistan (purple). Turkmenistan's percent of the USSR total methane emissions is shown in pink.

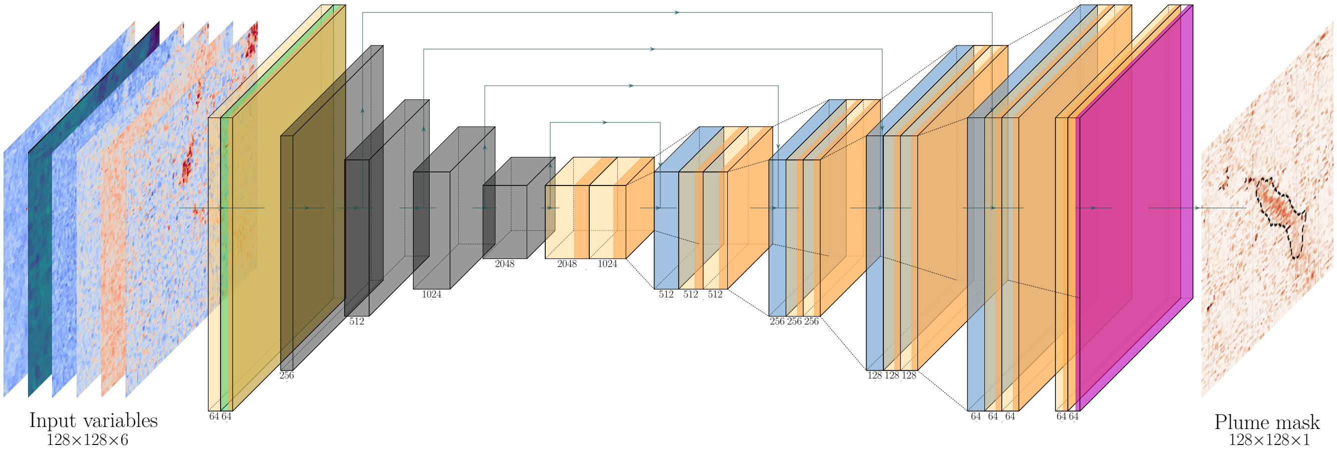

Fig. S2. Schematic diagram of the U-net model architecture. Grey boxes are the ResNeXt-50 (32×4d) pretrained model blocks. Light orange and light blue boxes represent convolutional layers and up-convolutional layers. The green box is batch normalization layer, dark orange layers are Rectified Linear Unit (ReLU) activation layers, and the last magenta layer is sigmoid activation layer.

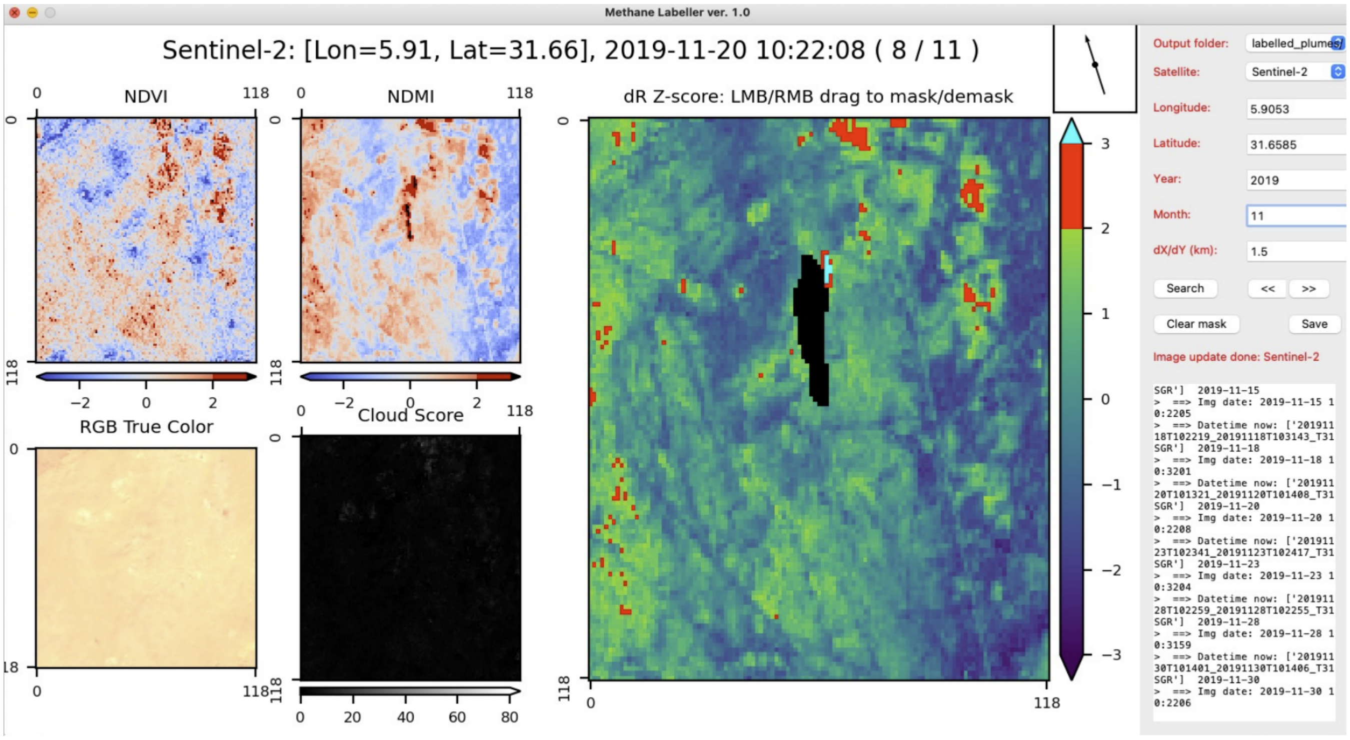

Fig. S3. Panel of the graphical user interface (GUI) for labelling methane plumes from the literature.

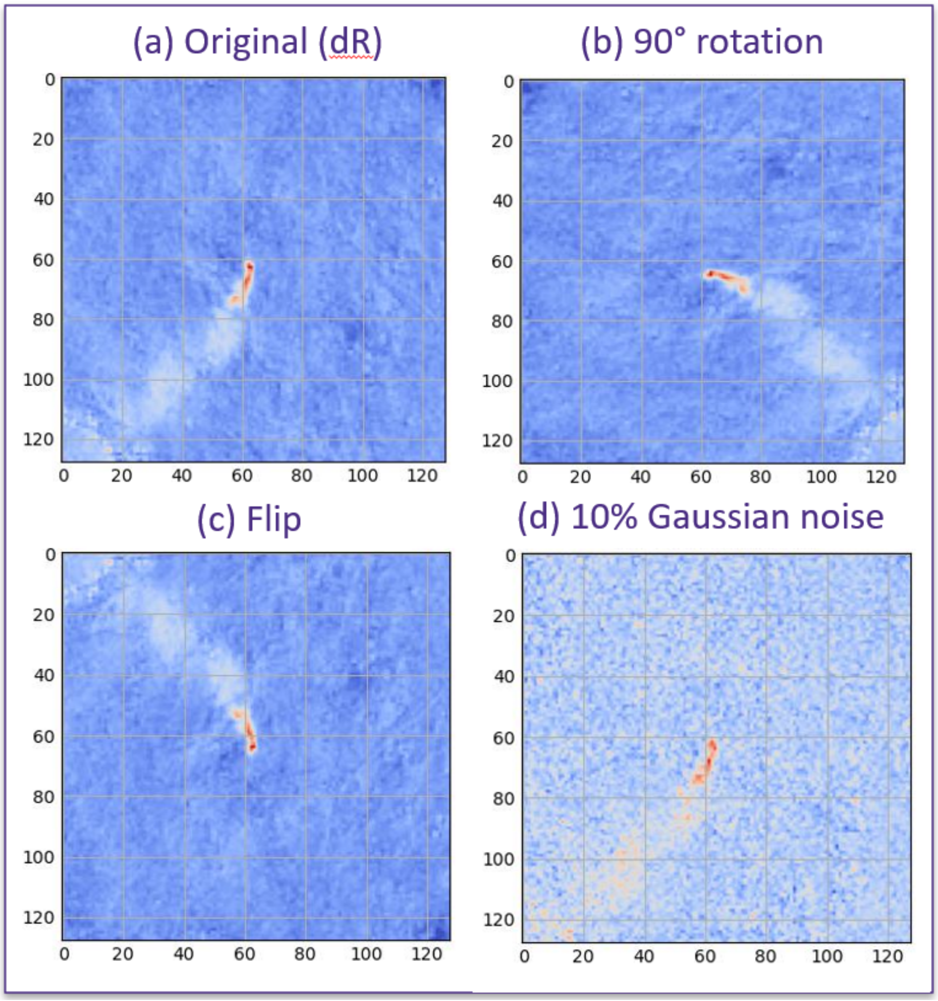

Fig. S4. Augmentation steps applied on the training data set.

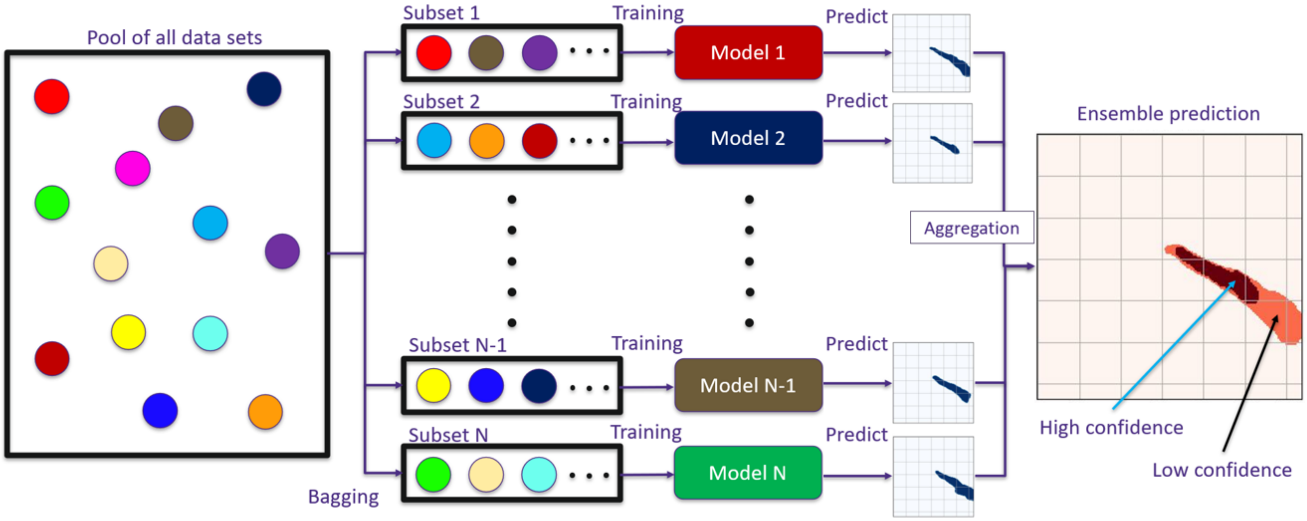

Fig. S5. Schematic diagram of the construction and the application of the ensemble of U-net models.

Table S1. Hyperparameters associated with training of each U-net model in the ensemble.

Ensemble number	Learning rate	Batch size	Number of epoch
1	1.55e-4	8	33
2	1.21e-4	10	26
3	1.26e-4	9	30
4	1.05e-4	10	25
5	1.97e-4	9	33
6	1.24e-4	14	27
7	1.58e-4	8	32
8	1.31e-4	12	27
9	1.49e-4	5	31
10	1.00e-4	5	27
11	1.53e-4	14	34
12	1.85e-4	14	30
13	1.28e-4	13	34
14	1.90e-4	12	33
15	8.68e-5	11	28
16	8.35e-5	6	31
17	9.98e-4	6	31
18	9.90e-5	5	33
19	1.82e-4	13	26
20	1.88e-4	13	31

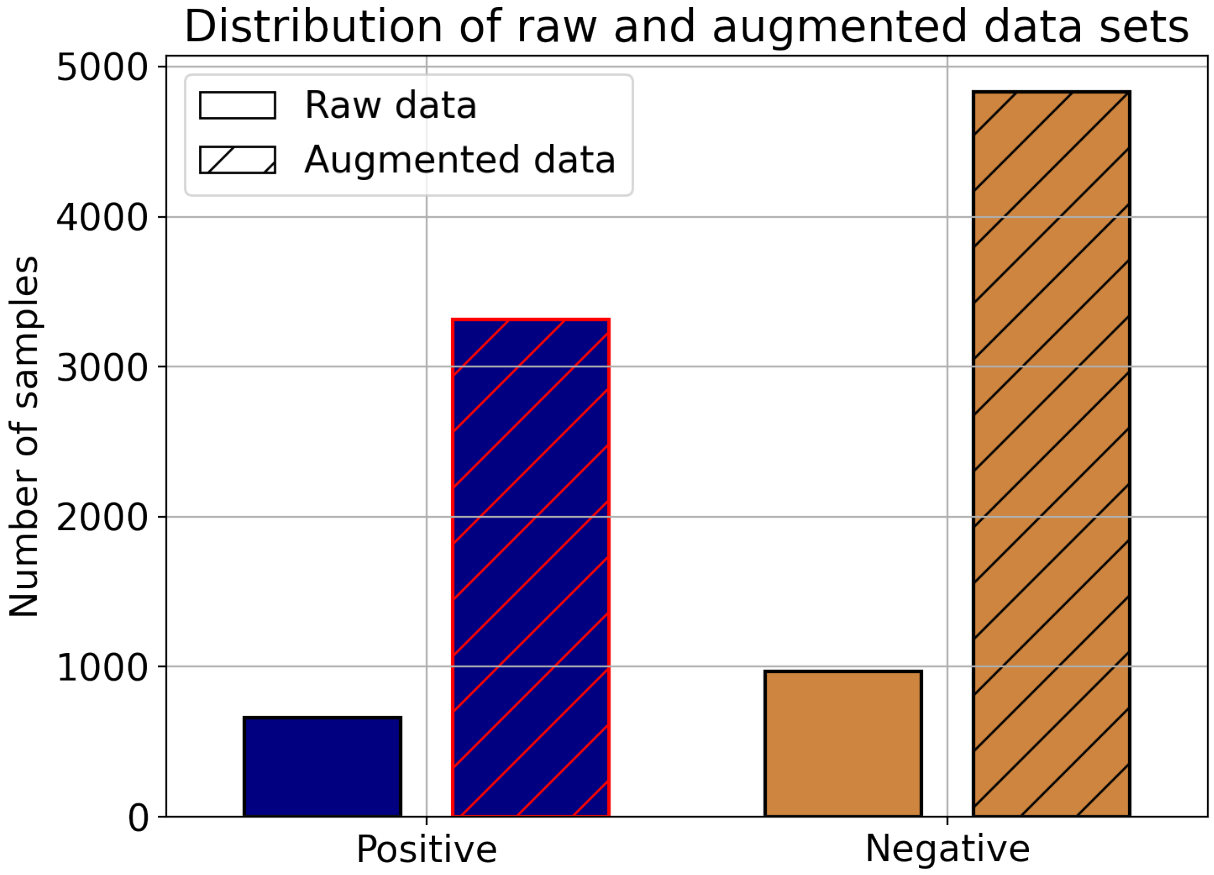

Fig. S6. Statistics about the original labelled data sets and the augmented data sets.

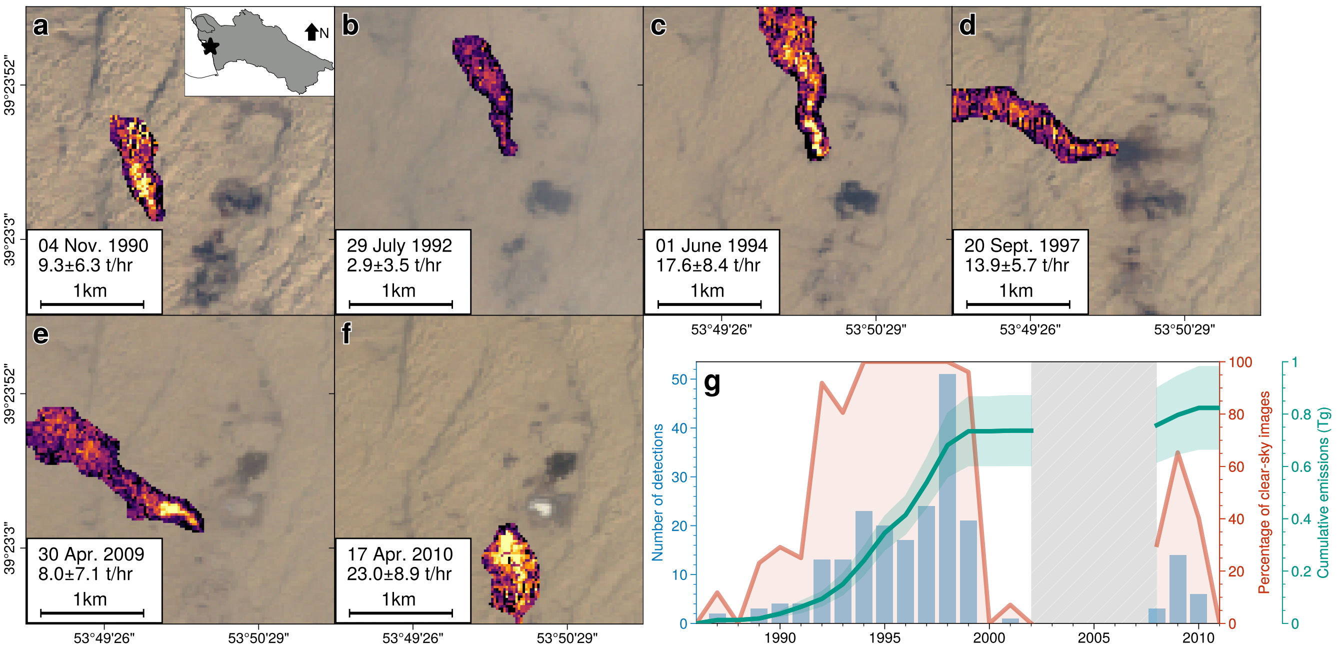

Fig. S7. Persistent regional methane emissions from the Barsagelmez Oil Field. (Panels a-f) Example methane plumes from the Barsagelmez Oil Field (39.391°N, 53.833°E) from 1986–2011. (Panel g) Number of detections (light blue), percent of clear-sky scenes with detections (orange), and estimated cumulative emissions from this source (teal). Gray shaded area indicates years with no Landsat 5 images available on Google Earth Engine due to the decentralized handling and distribution of Landsat 5 data sets.

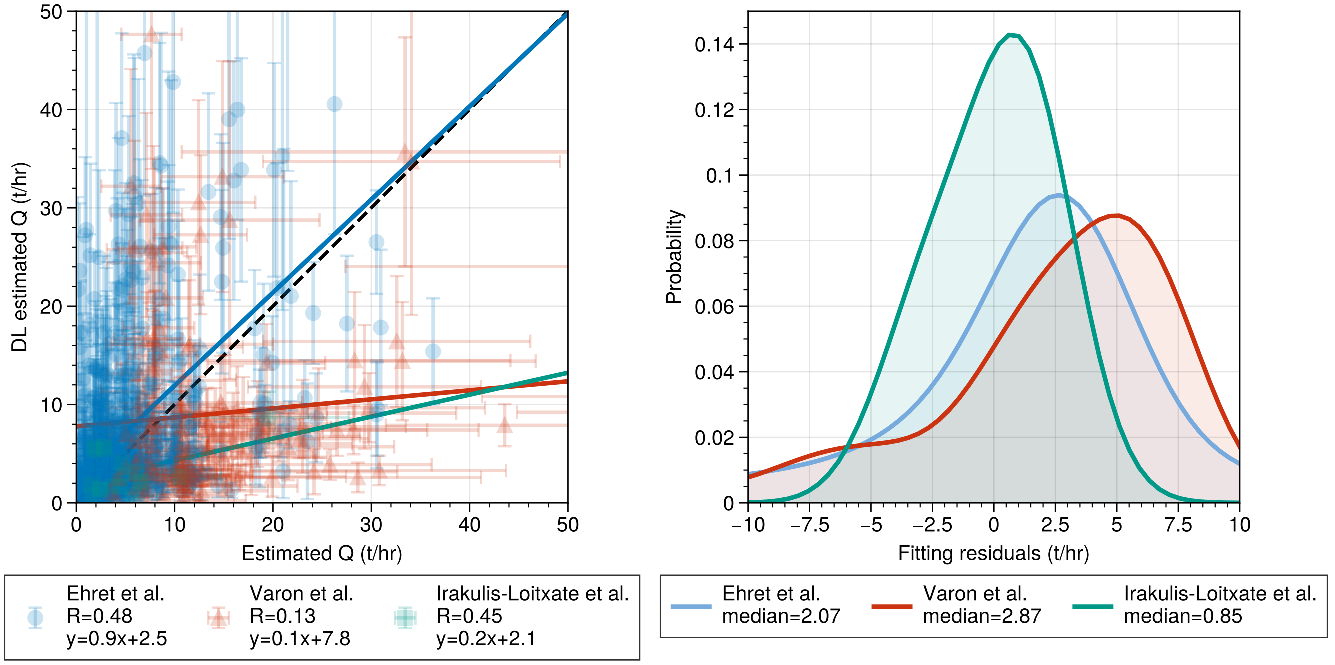

Fig. S8. Validation of the flux rates estimated using our system and other studies from the literature. (a) shows the correlation between the reported flux rates from (1–3) and our corresponding estimates. (b) shows the distribution of the residuals from linear regression in (a).

References

1. T Ehret, et al., Global tracking and quantification of oil and gas methane emissions from recurrent sentinel-2 imagery. *Environ. Sci. & Technol.* **56**, 10517–10529 (2022) PMID: 35797726.
2. DJ Varon, et al., Quantifying methane point sources from fine-scale satellite observations of atmospheric methane plumes. *Atmospheric Meas. Tech.* **11**, 5673–5686 (2018).
3. I Irakulis-Loitxate, et al., Satellite-based survey of extreme methane emissions in the permian basin. *Sci. Adv.* **7**, eabf4507 (2021).
